# Carbon Nanotube Ink Dispersed by Chitin Nanocrystals for Thermoelectric Converter for Self‐Powering Multifunctional Wearable Electronics

**DOI:** 10.1002/advs.202204675

**Published:** 2022-10-06

**Authors:** Yunqing He, Xiaoying Lin, Yue Feng, Binghong Luo, Mingxian Liu

**Affiliations:** ^1^ Department of Materials Science and Engineering College of Chemistry and Materials Science Jinan University Guangzhou 511443 P. R. China

**Keywords:** biocompatibility, chitin nanocrystals, multiwalled carbon nanotube, self‐powering wearable sensing, thermoelectric generator

## Abstract

The screen‐printing process of conductive ink can realize simple and large‐scale manufacture of micro/nano patterns for producing wearable electronic products. Herein, chitin nanocrystals (ChNCs) are used as a dispersant for the preparation of multiwalled carbon nanotube (MWCNT) ink with high viscosity and uniformity by ultrasound treatment. ChNCs can interact with MWCNT in noncovalent ways, including *π*–*π* and hydrophobic interactions. ChNCs/MWCNT (CCNT) ink does not aggregate even after standing for 3 months with a maximum MWCNT concentration of 33 mg mL^−1^ and dispersion efficiency of 91.1%. Using CCNT ink, a paper‐based thermoelectric generator (TEG) is manufactured by screen‐printing technology. With good thermoelectric and strain sensing properties, CCNT coated paper can stably collect human energy at room temperature to realize self‐powering. The CCNT coated paper‐based TEG can convert thermal voltage signals into musical notes, monitor the changes in human behavior and respiratory rate, and monitor joint movements. Moreover, CCNT coated paper has no cytotoxicity by CCK‐8 and live/dead staining. This work puts forward a strategy of green preparation of MWCNT‐based ink by adding renewable chitin, which opens up a new way to apply MWCNT‐based ink in self‐powering wearable multifunctional sensors.

## Graphical abstract

A stable and biocompatible multiwalled carbon nanotube ink stabilized by chitin nanocrystals is prepared, in which the two nanoparticles can interact via *π*–*π* and hydrophobic interactions. The conductive ink is coated on paper by screen‐printing technology to obtain the paper‐based thermoelectric generator, which shows multifunctional applications in self‐powering wearable sensor.

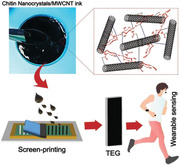

## 1 Introduction

Conductive inks can conveniently fabricate multifunctional electronic devices by the printing process, such as printing sensors,^[^ ^1^, ^2^ ^]^ generators,^[^ ^3^ ^]^ energy storage devices,^[^ ^1^ ^]^ and various flexible wearable electronic products.^[^ ^4^ ^]^ The selection of conductive ink is the key part of flexible electronics' product characteristics. Generally, the conductive ink should meet the requirements of good conductivity, processability, stability, printability, adhesion, and bending resistance of the printed coating.^[^ ^5^, ^6^ ^]^ With the rapid development of wearable electronics and bioelectronics, and the enhancement of environmental protection concepts, water‐based conductive “green” ink with proper rheology and good biocompatibility has gradually become the research focus.

The conductive solid particles in ink mainly include metal materials (metal nanoparticles and nanowires) and carbon‐based materials (conductive carbon black, carbon nanotube (CNT), graphene, and carbon quantum dots).^[^ ^7^ ^]^ Among them, CNT is considered an ideal candidate material for high‐quality conductive ink, which has unique electrical and thermal characteristics, high stability, and low density.^[^ ^5^, ^6^, ^7^, ^8^ ^]^ A majority of the researches on conductive ink were focused on single‐walled carbon nanotubes, while there are few reports on multiwall CNT (MWCNT). It is due to the single‐walled CNT having higher uniformity and conductivity.^[^ ^9^ ^]^ However, MWCNT is cheaper, more convenient to produce, more flexible, and better controlled in shape, which makes it easier to realize mass production. Unfortunately, the intrinsic strong *π*–*π* interaction among the tubes makes it easy to self‐aggregate in water, which greatly affects the uniformity, shelf‐life, and processability of CNT‐based conductive ink.^[^ ^10^ ^]^ Many methods have been developed to solve the aggregation problem of CNT dispersion in water. Chemical modification^[^ ^11^ ^]^ and surfactant^[^ ^12^ ^]^ have been widely used to change the surface characteristics of CNT, thus improving the dispersibility of CNT in water. However, the surface structure of CNT may be damaged by these treatments, and the conductivity becomes deterioration.^[^ ^6^ ^]^ What's more, these methods harm the biocompatibility of the obtained CNT‐based ink.^[^ ^8^ ^]^

To improve the biocompatibility of CNT, many natural biological polysaccharides and proteins have been used to disperse CNT via noncovalent interactions. For example, Li et al.^[^ ^9^ ^]^ prepared a water‐based composite ink with excellent stability by using carboxylated nanocellulose as a dispersant of MWCNT, which significantly improved the thermoelectric conversion efficiency of the coating. Wang et al.^[^ ^13^ ^]^ developed MWCNT‐based conductive ink with highly dispersed mediated by carboxymethyl xylan hydrate crystals, which have excellent conductivity. Liang et al.^[^ ^8^ ^]^ designed a green way to prepare highly stable and biocompatible CNT conductive ink mediated by sericin, which also has excellent conductivity. Feng et al.^[^ ^5^ ^]^ prepared a single‐walled carbon nanotube ink highly dispersed by sulfated nanocellulose, and the dispersion limit reached 80 wt%. The aforementioned natural materials can increase the dispersion ability of CNTs in water due to the interactions of *π*–*π* interactions and/or hydrophobic interactions. Therefore, natural macromolecules have great potential in the field of CNT dispersions. Natural materials have advantages of rich content and good biocompatibility, so their assisted dispersed inks have great potential in fabricating wearable electronic devices. For example, Duan et al.^[^ ^6^ ^]^ prepared a CNT‐based ink using cellulose derivatives as the solvent exhibited excellent sensing performance as wearable strain sensors. Lin et al.^[^ ^10^ ^]^ designed a hydrogen bond cross‐linked network based on carboxylic styrene butadiene rubber and sericin noncovalently modified CNT, making it a wearable multifunctional sensor for detecting human body movement and skin temperature.

Chitin nanocrystals (ChNCs) are natural biological nanomaterials^[^ ^14^ ^]^ with a one‐dimensional rod‐like structure, which can be easily extracted from shrimp and crab shells. Similar to cellulose nanocrystals, ChNCs are nontoxic, highly biodegradable, and colloidally stable.^[^ ^15^ ^]^ With abundant acetylamino groups and a small number of amino groups in its molecular chain, ChNCs are positively charged. CNT is always negatively charged, which makes ChNCs have a high affinity with CNT. Additionally, the molecular chain of ChNCs is amphiphilic, which can be stably arranged on the surfaces of hydrophobic CNT by hydrophobic action. The hydrophilic end of ChNCs reduces the surface tension outward and prevents the association of CNT by steric hindrance. Moreover, previous research has proved that ChNCs is a green adhesive,^[^ ^16^ ^]^ so it can fix conductive fillers on various substrates (such as paper, glass, and PET) well. As far as we know, there is no report on ChNCs as a dispersant and binder for CNT. Besides, compared with other natural dispersants (such as sulfated nanocellulose, carboxylated nanocellulose fiber, nanoscale xylan hydrate crystal, and sericin), ChNCs exhibit positively charged surfaces, and display better thermal stability, antibacterial property, biosafety, and abundance of resource.

Wearable electronic devices have attracted extensive research attentions because of their convenience and flexibility.^[^ ^17^ ^]^ They are widely used in daily life, such as health monitoring, sports, and entertainment.^[^ ^18^ ^]^ However, traditional electronic devices often have an external power supply and frequent battery replacement, which bring inconveniences and even potential safety hazards to humans.^[^ ^19^ ^]^ Therefore, developing a safe and stable self‐powering sensing system that can collect energy from the living environment or human body without an external power supply has become a research hotspot.^[^ ^20^ ^]^ Recently, energy harvesting technologies mainly include piezoelectric,^[^ ^21^ ^]^ triboelectric,^[^ ^22^ ^]^ thermoelectric,^[^ ^23^ ^]^ and photovoltaic.^[^ ^24^ ^]^ The thermoelectric material can directly convert heat energy into electric energy by using the temperature difference between the human body and the environment.^[^ ^23^ ^]^ As a kind of green energy, thermoelectric has attracted wide attentions because of its safety and environmental‐friendly.^[^ ^9^ ^]^ At present, many achievements have been made in the research of self‐powering sensors based on thermoelectric. For example, He et al.^[^ ^17^ ^]^ designed a thermoelectric fabric based on CNT/polyvinylpyrrolidone (PVP)/polyurethane composite, which can realize language conversion, monitor breathing, and detect joint movement by using human body temperature. The prepared multifunctional self‐powering sensor can sensitively detect temperature and strain.^[^ ^25^ ^]^ Zhao et al.^[^ ^18^ ^]^ reported a transparent paper‐based thermoelectric generator (TEG) using Bi_0.5_Sb_1.5_Te_3_ and Bi_2_Se_0.3_Te_2.7_ for human waste heat recovery. Dong et al.^[^ ^26^ ^]^ prepared flexible paper‐based TEG using Bi_2_Te_3_ and Sb_2_Te_3_ by simple vacuum filtration method, which has high heat sensing. Paper is mainly made of cellulose fiber, and cellulose is the most abundant natural polysaccharide on earth.^[^ ^25^ ^]^ Recently, the paper has become a popular and potential flexible electronics element^[^ ^27^ ^]^ because of its foldability, biosafety, biodegradability, low thermal conductivity, and low density. Moreover, the paper has excellent penetrability, a three‐dimensional network structure of randomly oriented cellulose fibers, and good thermal stability.^[^ ^28^ ^]^ Therefore, cellulose paper may be an ideal substrate for flexible TEG.

In this work, MWCNT water‐based composite ink with excellent uniform and stable dispersion was prepared with ChNCs as dispersant and adhesive by ultrasonic treatment. We coated the ink on paper by screen‐printing technology to obtain the paper‐based TEG, showing the multifunctional applications of ChNCs/MWCNT (CCNT) ink. The paper‐based TEG had finger temperature response, in which thermal voltage signals could be converted into musical notes. In addition, the TEG could be attached to the skin to monitor the body movement, integrated on a mask to monitor breathing, and successfully detected the bending movement of the finger joints under the self‐powering condition. The L929 cell experiment verified that CCNT coated paper‐based TEG had good biocompatibility, ensuring its application safety. The environmental‐friendly ChNCs assisted MWCNT ink showed the promising application in paper‐based TEG, which promoted the development of “green” ink in the field of self‐powering multifunctional wearable electronics.

## 2 Results and Discussion

### 2.1 Preparation and Characterization of the CCNT Dispersions

**Figure 1:**
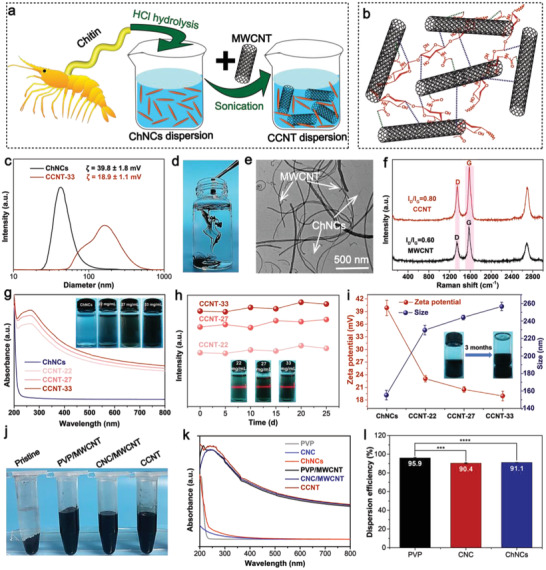
a) Schematic illustration of the formation of CCNT dispersion. b) Noncovalent interactions between MWCNT and ChNCs. c) Zeta potentials and size of ChNCs and CCNT‐33. d) Drops of sonicated CCNT in water. e) TheTEM image of CCNT dispersion. f) Raman spectra of MWCNT and CCNT powder. g) UV–vis absorption spectra, h) the maximum UV‐vis absorbance (260 nm), and i) zeta potential and size of ChNCs and CCNT dispersions. j) Photograph of MWCNT‐water mixtures without dispersant and with 40 mg L^−1^ of different dispersants. k) UV–vis spectra of PVP, cellulose nanocrystals (CNC), ChNCs, PVP/MWCNT, CNC/MWCNT, and CCNT. l) Dispersion efficiency of PVP, CNC, and ChNCs. (***p = 0.0002, ****p < 0.0001, and *n* = 3. Data were analyzed by one‐way ANOVA and expressed as mean ± SD).

**Figure** **1**a shows the preparation process of CCNT dispersion. Briefly, a stable aqueous dispersion of ChNCs was obtained via hydrochloric acid hydrolysis of chitin. Then the stable CCNT dispersion was prepared by adding MWCNT and ultrasonic treatment. The successful dispersion of MWCNT in water was mainly due to the *π*–*π* interaction between ChNCs and MWCNT surface (Figure 1b). In addition, the coexistence of hydrophobic and hydrophilic groups in ChNCs makes them amphiphilic, and it can reduce the surface energy and acts as the surfactant in the aqueous phase. Hydrophobic–hydrophobic interaction occurred between ChNCs and MWCNT, thus further stabilizing the dispersion ability of MWCNT in water. Besides, there were electrostatic interactions between MWCNT and ChNCs. The Zeta potential of MWCNT is negative,^[^ ^29^ ^]^ while ChNCs is positively charged. Coulomb attractions can occur between them. As expected, the zeta potential of ChNCs decreased after the incorporation of MWCNT (Figure 1c). Nevertheless, the potential of CCNT dispersion could make MWCNT disperse stably in the water phase. Figure 1d shows that CCNT dispersion droplets could quickly diffuse in water without any visible aggregation, indicating that CCNT has good dispersibility in water. In contrast, the droplets of the ultrasonically treated MWCNT‐water mixture without ChNCs quickly sank into the water, resulting in a visible precipitate (Figure S1, Supporting Information). The dispersion degree of MWCNT in water was further evaluated by optical microscope and transmission electron microscopy (TEM). After standing for 5 days, MWCNT without dispersant had macrosedimentation (Figure S2, Supporting Information) and microagglomeration (Figures S2 and S3, Supporting Information), while MWCNT dispersed by ChNCs contained only a tiny amount of MWCNT aggregates (Figure S2, Supporting Information; Figure 1e). From the TEM image, MWCNT dispersed by ChNCs still retained a complete tubular structure, which indicates that the addition of ChNCs does not significantly change the structure of CNT.

Raman spectroscopy was used for analysis the structure change of MWCNT, and the results were shown in Figure 1f. Defects and curvature usually cause the D peak at about 1342 cm^−1^ in the nanotube lattice, and the G peak at about 1588 cm^−1^ represents the in‐plane vibration of the C− bond.^[^ ^30^ ^]^ Generally, *I*_D_/*I*_G_ is used to evaluate the defect degree of carbon materials, which is the critical structural factor in determining the interface strength between ChNCs and MWCNT.^[^ ^31^ ^]^ Compared with MWCNT, the *I*_D_/*I*_G_ of CCNT increased from 0.60 to 0.80, indicating that noncovalent interactions were formed between ChNCs and MWCNT, leading to more defects. The noncovalent interaction was further confirmed by Fourier transform infrared (FTIR) (Figure S4, Supporting Information). Characteristic absorption peaks of ChNCs were observed at 3335, 3262, 1652, and 1554 cm^−1^, which were attributed to O−H stretching, N−H stretching, amide I, and amide II, respectively. There was no characteristic peak in MWCNT sample, which indicated that there were no oxygen‐containing groups on the surfaces of MWCNT. It was found that there was no new absorption peak in the infrared spectrum of CCNT by adding ChNCs to MWCNT. However, the absorption peak's intensity became weak, suggesting that ChNCs and MWCNT had a non‐covalent bond.

### 2.2 Dispersion Limits and Performance of CCNT Dispersions

The UV‐vis absorption spectrum was used to estimate the dispersion limit of MWCNT by ChNCs. Figure 1g shows that the higher the dispersion concentration, the stronger the absorbance, and the dispersion limit of MWCNT could reach 33 mg mL^−1^. If the MWCNTs dispersion concentration is beyond to 33 mg mL^−1^, the absorbance value will not increase, suggesting the dispersion limit was achieved. Thermogravimetry (TG) analysis was used to determine the maximum amount and thermal stability of MWCNT dispersed by ChNCs. Figure S5 (Supporting Information) shows the TG and derivative thermogravimetric (DTG) curves of MWCNT, ChNCs, and CCNT (33 mg mL^−1^). The TG and DTG curves of MWCNT were almost a straight line, and the weight loss at 800 °C was about 5%, which showed excellent thermal stability. The DTG decomposition peak temperature of ChNCs was 353.8 °C which was much lower than that of CCNT (393.8 °C), indicating that noncovalent interactions between ChNCs and MWCNT enhanced the thermal stability of CCNT. The residues of MWCNT, ChNCs, and CCNT at 800 °C were 95.2%, 2.5%, and 44.8%, respectively. As TG results strongly depend on residual carbon and sample structure, the calculated MWCNT content of CCNT based on TG results was close to the actual value (the weight ratio of ChNCs: MWCNT was 11:9).^[^ ^32^ ^]^

To evaluate the stability of CCNT dispersion, CCNT dispersion was allowed to stand for 25 days, and the maximum absorbance of CCNT dispersion was tested regularly. Figure 1h shows that the absorbance of CCNT with different dispersion concentrations at 260 nm had no obvious change and showed the typical Tyndall effect, indicating that CCNT dispersion had uniformity and stability. The average particle size and Zeta potential of CCNT dispersions with different dispersion concentrations were then measured. Compared with pure ChNCs dispersion, the average particle size of CCNT dispersion increased, and the Zeta potential decreased (Figure 1i). Dynamic light scattering measured the hydrodynamic diameter of the different dispersion. Part of ChNCs might adhere to MWCNT,^[^ ^33^ ^]^ which led to the increase of the average particle size of CCNT. The Zeta potential of CCNT was about +18.9 mV, which was about half lower than that of pure ChNCs (+39.8 mV). Interestingly, there was no severe aggregation of CCNT dispersion after standing for 3 months, because the good colloidal property of ChNCs help stabilize MWCNT.^[^ ^13^ ^]^ Up to now, the ink can be stably stored for more than 9 months, and no evident precipitation or particle aggregation was observed.

We also compared the performance of ChNCs with other dispersants. PVP is one of the most commonly used commercial dispersants for CNT, and natural CNC is also an effective dispersant for CNT. Therefore, PVP and CNC were selected to represent macromolecular surfactant and nanoscale polysaccharide, respectively. The same mass of MWCNT was dispersed in the same concentration of dispersants and then removed the undispersed MWCNT by centrifugation to obtain MWCNT dispersions (Figure 1j). The UV–vis absorption spectra of dispersants and MWCNT dispersions were shown in Figure 1k. The typical absorption peak of MWCNT dispersions appear at 200–300 nm,^[^ ^34^ ^]^ we chose the absorbance of MWCNT dispersions at 260 nm for calculation. Based on the results of UV–vis spectra, the concentration of MWCNT in the dispersions after centrifugation was calculated. The results showed that the dispersion efficiency of ChNCs was about 91.1%, which was higher than that of CNC (90.4%) (Figure 1l). The excellent dispersion ability of ChNCs for MWCNT was mainly attributed to two aspects. On the one hand, ChNCs suspension exhibits good colloidal stability. On the other hand, noncovalent interactions occur between ChNCs and MWCNT. Being a common commercial dispersant for CNT, PVP (95.9%) had a higher dispersion efficiency than ChNCs (91.1%), which was acceptable. However, there are potential safety hazards in PVP, and the monomer produced by PVP degradation is harmful to the human being.^[^ ^35^ ^]^ ChNCs is the second largest natural polysaccharide with safety and biodegradability, so it can be a good substitute for PVP.

### 2.3 Characterization of CCNT‐Based Conductive Ink

**Figure 2:**
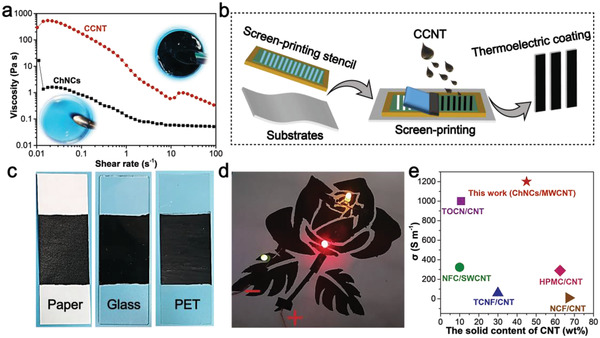
a) Viscosity vs shear rate for ChNCs and CCNT. b) Schematic of the screen‐printing process. c) Photographs of CCNT ink printed on paper, glass, and PET substrates. d) Conductivity of CCNT ink. e) Comparison in electrical conductivity (σ) of CNT with different natural dispersant (TEMPO‐oxidized cellulose nanofiber (TOCN), hydroxylpropyl methyl cellulose (HPMC), nanofibrillated cellulose (NFC), nanocellulose extracted from tunicate (TCNF), nanofibrillar cellulose (NCF)).^[^ ^6^, ^36^ ^]^

Due to the excellent colloidal stability and uniformity, the possibility of CCNT dispersion with MWCNT content of 33 mg mL^−1^ as conductive ink was studied. A rheological property test was conducted at room temperature to clarify the characteristics of CCNT ink, as shown in **Figure** **2**a and Figure S6 (Supporting Information). Figure 2a shows the change of apparent viscosity of ChNCs and CCNT with shear rate. ChNCs and CCNT are non‐Newtonian fluids with shear thinning characteristics.^[^ ^37^ ^]^ However, the viscosity of ChNCs was much lower than that of CCNT, which indicated that MWCNT improved the viscosity of ChNCs because MWCNT has a high aspect ratio and interactions occur between ChNCs and MWCNT. The inset in Figure 2a shows the appearance of ChNCs and CCNT dispersion. ChNCs was a transparent dilute fluid, while CCNT was a high viscosity fluid. The storage modulus (*G*′) and loss modulus (*G*″) of CCNT inks were measured to evaluate their viscoelasticity, as shown in Figure S6 (Supporting Information). The platform of *G*′ of ChNCs was between 10^1^ and 10^2^ Pa, which was about an order of magnitude higher than that of *G*″, which further verified that ChNCs are elastic solid.^[^ ^38^ ^]^ The modulus of CCNT was about one order of magnitude higher than that of ChNCs, which indicated that MWCNT enhanced the network of ChNCs. All these results proved that CCNT ink may show good printability.

The contact angles of the dispersion on paper (0°), glass (42.9°), and PET (80.8°) substrates were tested to explore the applicability of CCNT ink printing on different substrates (Figure S7a, Supporting Information). All the contact angles of CCNT dispersions on these substrates were less than 90°, which indicated good wettability.^[^ ^39^ ^]^ The paper‐based sensor was prepared by screen‐printing techniques (Figure 2b). The ink was further printed on glass and PET film to verify the extensive printing compatibility of the ink on different substrates (Figure 2c). Additionally, the printed CCNT coating can be bent on a flexible substrate (paper or PET) (Figure S7b, Supporting Information). For further verification of the applicability of the ink to conductive electronic circuits, three LED lamps with different colors were connected to the screen‐printed pattern based on CCNT ink (Figure 2d). With a circuit voltage of 9.0 V, the three LED lamps showed bright light simultaneously, indicating that the ink's printed conductive pattern can be used in electronic circuits. It is worth noting that CCNT ink could be well redispersed after freeze‐drying (Figure S8, Supporting Information) because of its hydrophilicity and dispersibility. It is convenient to adjust ink concentration and is beneficial to storage, transportation, and recycling in commercial applications. Owing to the existence of ChNCs, the CCNT dispersion can form a flexible self‐supporting nanocomposite film after drying (Figure S9, Supporting Information). Compared with other dispersants, the MWCNT dispersed by ChNCs had a better σ (1150 S m^−1^) (Figure 2e), indicating that ChNCs could disperse MWCNT uniformly. In a word, these results showed that CCNT dispersion is a potential conductive printable ink. Based on the good conductivity, MWCNT has a thermoelectric effect, which refers to the phenomenon that electrons in heated MWCNT move from high temperature region to low temperature region with the temperature gradient, resulting in current or charge accumulation.^[^ ^9^ ^]^ Therefore, CCNT ink can be printed on a flexible substrate (such as cellulose paper) by screen printing to collect the heat of the human body and prepare a self‐powering wearable device.

### 2.4 Biocompatibility of CCNT Coated Paper

Many traditional sensors are discarded in the environment, which harms the ecological environment. This study used wheat as a plant model to evaluate the ecotoxicity of the CCNT coated paper based sensor. It was found that there was no apparent difference in appearance between wheat cultured on paper containing CCNT coating and the control group after culture for 10 days (Figure S10a, Supporting Information). Moreover, the survival rate of wheat seedlings reached 100%, and the growth height and root length were close to those of the control group (Figure S10b, Supporting Information). Experiments on aquatic wheat showed that CCNT coated paper does not affect the growth of plants.

As a sensor, the coated paper will inevitably come into contact with human skin, so it is necessary to evaluate its cytotoxicity. According to previous studies on skin safety, we chose mouse fibroblast line L929 as the in vitro model.^[^ ^16^ ^]^ The cytotoxicity of coated paper was evaluated by the viability of L929 cells incubated with CCNT coated paper extracts of different concentrations for 24 or 48 h. In Figure S10c (Supporting Information), the cell viability of the sample groups was all close to 100%, indicating that the extraction solution had no toxicity to the cells. The toxicity of coated paper extract to L929 cells was further verified by live/dead staining. Figure S10d (Supporting Information) shows that L929 cells showed green fluorescence after incubation with different concentrations of extracts for 24 or 48 h, and almost no red color was observed, indicating no dead cells. Furthermore, there was no significant difference in cell density between the sample group and the control group, and the number of L929 cells incubated for 48 h increased significantly. The results of live/dead staining were consistent with those of CCK‐8, which indicated that the extraction solution of the CCNT coating had good biocompatibility and safety. Additionally, paper, ChNCs, and MWCNT are all environmental‐friendly and harmless to the human body.^[^ ^6^, ^16^ ^]^ Therefore, based on the good conductivity of CCNT coating, printing CCNT ink on cellulose paper has the potential to manufacture green self‐powering sensors.

### 2.5 Characterization of CCNT Paper Based TEG

**Figure 3:**
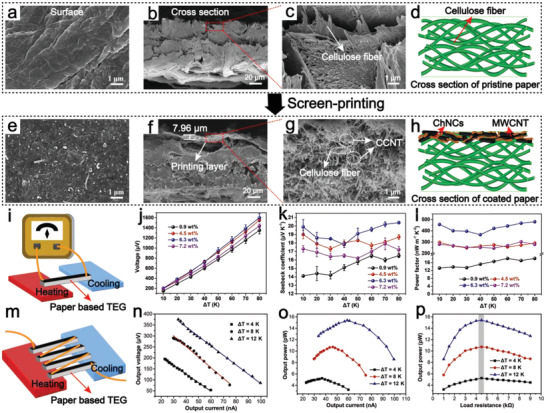
Surface SEM images of a) the pristine paper and e) CCNT coated paper; cross‐sectional SEM images of b,c) the pristine paper and f,g) CCNT coated paper. Schematic diagram of d) the pristine paper and h) CCNT coated paper. i) Schematic diagram of an open‐circuit voltage measurement device of CCNT coated paper based TEG. j) Voltage, k) Seebeck coefficient, and l) PF of TEG coating prepared by different amounts of CCNT. m) Schematic diagram of a TEG device composed of four CCNT coated paper connected in series. Relationship between n) the output voltage and o) output power versus the current of the TEG under different Δ*T*. p) Output power of the TEG as a function of load resistance.

The paper coating requires conductive dispersion to have high solid content and uniformity. MWCNT dispersion assisted by ChNCs with superior dispersion can be used as a conductive coating for the electrical functionalization of paper. **Figure** **3**a,e shows the surface appearance of pristine paper and coated paper, respectively. It can be seen that the coating covered the fibril skeleton, and no apparent aggregation and agglomeration can be observed, indicating that MWCNT was tightly and effectively fixed on the cellulose surface. Figure 3b,c shows cross‐sectional images of pristine paper, while Figure 3d provides a cross‐sectional microscopic model. The paper comprised adjacent and interwoven cellulose fibers. Figure 3f shows a cross‐sectional scanning electron microscope (SEM) image of CCNT coated paper. It can be seen that a dense layer was formed with a thickness of about 8 µm on the paper surface, which was enlarged in Figure 3g. It could be seen that CCNT ink not only adhered to the paper's surface but also partially penetrated the upper internal layers of the paper. The coating was uniformly attached to the cellulose fiber, forming a dense layer on the surface and inside so that the cellulose fiber could not be observed. The final coated paper consisted of the surface and the upper internal adhesive layer, and its structural model was shown in Figure 3h. CCNT ink formed dense layers in the paper adjacent to each other, forming conductive paths. Uniform MWCNT dispersion is significant for the high conductivity of paper. Our previous research suggested that ChNCs are a kind of green water based adhesive,^[^ ^16^ ^]^ so ChNCs not only promote the dispersion of MWCNT uniformly, but also make MWCNT tightly bonded to cellulose fibers. Hence, the conductive coating has uniform stability.

To explore the influence of different concentrations of CCNT coating on the thermoelectric properties of coated paper, we connected the coated paper to a homemade test platform for testing (Figure 3i). The same process obtained thermoelectric paper, and its output voltage, Seebeck coefficient, and power factor (PF) were tested and compared (Figure 3j–l). Figure 3j shows the output voltage of coatings with different concentrations under different temperature differences. It can be seen that the output voltage obtained by coating 6.3 wt% CCNT was the highest, reaching 1600 µV at a temperature difference of 80 K. However, the output voltage reduced to 1437 µV as the coating concentrations continuously increased to 7.2 wt%. Perhaps the main reason was that the high‐concentration ink had high viscosity, which made it difficult for the ink to be spread out in the printing process, resulting in flocculation of some MWCNT. Finally, the thermoelectric performance of coated paper decreased. The variation of Seebeck coefficient of coatings with different concentrations under different temperature differences was shown in Figure 3k. Seebeck coefficient is an important parameter to characterize the conversion efficiency of thermoelectric energy, which is defined as the magnitude of thermoelectric voltage generated per degree of temperature difference. The MWCNT dominated the Seebeck coefficient of CCNT paper based TEG, so Seebeck coefficient remained stable in the same concentration of coated thermoelectric paper. Interestingly, there was a slight fluctuation of Seebeck coefficient at different temperature differences, which may be due to the unstable indoor airflow leading to heat loss of thermoelectric paper. The concentration and dispersion of CCNT had great influence on the *σ* of the coated paper. When the concentration of CCNT coating increased from 0.9 to 6.3 wt%, the *σ* of coated paper also increased correspondingly (Figure S11, Supporting Information). However, when the concentration of CCNT ink further increased to 7.2 wt%, *σ* decreased due to the aggregation of MWCNT at high concentration. The PF of the material is related to *σ* and Seebeck coefficient (*S*) (PF = *σS* ^2^), and a higher PF means that more energy is generated at the same temperature difference. The changing trend of PF shown in Figure 3l was similar to that of Seebeck coefficient, which indicated that the thermoelectric properties of the prepared coated paper strongly depended on the coating amount and dispersion degree of MWCNT. As the 6.3 wt% CCNT coated paper showed the highest PF, it was selected as the optimal concentration for subsequent testing. To estimate thermoelectric figure of merit (ZT) value, the thermal conductivity of CCNT coated paper was calculated as 0.032 W m^−1^K^−1^ by the flash system, indicating that the CCNT coated paper had low thermal conductivity.^[^ ^9^ ^]^ The thermoelectric performance of CCNT coated paper was further evaluated by the ZT (ZT = *S* ^2^ *σTκ* ^−1^, where *S* is the Seebeck coefficient, *σ* is the electrical conductivity, *T* is the temperature, and *κ* is the thermal conductivity). The ZT value of CCNT coated paper was 0.0041, which was higher than reported research on CNT‐based thermoelectric materials (Table S1, Supporting Information).^[^ ^40^, ^41^ ^]^ It is noticed that irrespective of the low PF of CCNT ink coated paper in this work, the ZT value was comparable to some wearable thermoelectric devices.^[^ ^18^, ^41^ ^]^ Therefore, the multiunit TEG was further fabricated using CCNT‐coated paper.

Owing to the simple printing process and low cost of thermoelectric coating, CCNT coated paper can be easily produced into flexible TEG on a large scale. The research set up a device composed of four 5 × 15 mm coated papers in series to further demonstrate its power generation capacity (Figure 3m). Measurement showed that the internal resistance of TEG was 4500 Ω. The TEG is equivalent to the power supply. By connecting the TEG with the external load resistor in series, the output voltage and output current curves under different temperature gradients were obtained, as shown in Figure 3n. The output voltage was inversely proportional to the current, and the output voltage increased with the increase in temperature difference. The *P*–*I* curve was obtained according to the output power (*P*) = *UI* (*U*, *I* are the corresponding output voltage and current, respectively) (Figure 3o). The maximum output power of TEG was 5.2, 10.7, and 15.4 pW when the temperature difference of 4, 8, and 12 K was applied, which was the same as the corresponding output power when the load resistance was 4500 Ω (Figure 3p). The aformentioned experimental data showed that when the load resistance was 4500 Ω, the output power of TEG reached the maximum value under all temperature differences, which was well matched with the internal resistance. Actually, TEG can be composed of hundreds to thousands of units. So, the power supply of microelectronic devices can be realized by connecting multiple coated papers in series and increasing the output voltage. In a word, CCNT coated paper prepared in this study showed good potential in self‐powering wearable electronic products.

### 2.6 Thermal Response Performance

**Figure 4:**
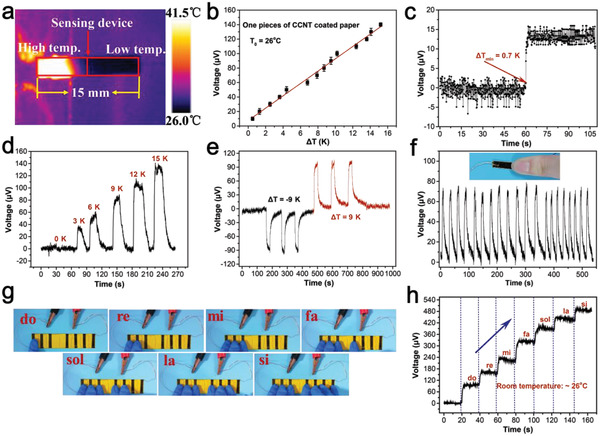
a) The infrared thermal image of the temperature distribution of a sensing device with one end heated, and the other end exposed to air. b) Measured output voltage as a function of Δ*T* (26 °C) at room temperature (*n* = 3). c) The minimum distinguishable Δ*T* of the output voltage signal of the sensor device. d) The output voltage response of the sensing device when Δ*T* is 3, 6, 9, 12, and 15 K, respectively. e) Measured sensing device response time under different heating–cooling cycles. f) Voltage response of one finger touch. g) Physical images of a self‐powering temperature sensor consisting of seven coated papers. h) Self‐powering temperature sensor corresponding to output voltage from one to seven finger touches.

The CCNT coated paper can be used as a self‐powering temperature sensor to detect the temperature change at room temperature based on the Seebeck effect of TEG. By adhering of conductive adhesive, two copper wires were directly attached to both ends of one piece of 5 × 15 mm CCNT coated paper and then connected to an electrometer for sensing test. The surface temperature distribution of TEG was recorded by an infrared camera at room temperature of 26 °C and relative humidity of 85%, as shown in **Figure** **4**a. There was a good linear relationship between the temperature difference between the two ends of coated paper and the output voltage at room temperature (*T*_0_ = 26 °C) (Figure 4b). Accordingly, temperature sensing could be realized by keeping the temperature of one end of coated paper and changing the temperature of the other end. Temperature resolution, response stability, and response time are important parameters to measure the performance of sensor equipment. Figure 4c shows that the minimum detectable temperature difference of TEG output voltage was 0.7 K. A slight temperature difference led to a noticeable voltage change of TEG, which indicated that the TEG had good temperature resolution.

The voltage generated by the TEG in the temperature difference range of 0 to 15 K was demonstrated in Figure 4d. With the increase of the temperature difference between TEG terminals, the generated voltage also increased. In Figure 4e, the output voltage of TEG under multiple cooling and heating cycles was further shown, which proved that the TEG had the potential to distinguish the stimulation of cold or heat sources continuously. The temperature response stability of TEG was characterized by the voltage response under finger touch (Figure 4f). Every cycle's maximum output voltage was the same because the temperature change caused by finger touch was nearly the same. It can be seen from Figure S12 (Supporting Information) that the temperature response time of TEG was 2.05 s and the recovery time was 4.92 s.

Based on the TEG's accurate temperature detection and identification ability, we designed a self‐powering sensor that converted thermal voltage signals into musical notes. The sensor consisted of seven pieces of 5 × 15 mm CCNT coated paper connected in series by copper wires. When different numbers of fingers touched the sensor, it generated different thermoelectric signals in response (Figure 4g,h). With the increase in the number of touching fingers, the thermal voltage signal of the sensor would increase significantly. Therefore, we defined the number of touching fingers “1”, “2”, “3”, “4”, “5”, “6”, and “7” as the notes “do”, “re”, “mi”, “fa”, “sol”, “la”, and “si”, respectively. On this basis, we showed some notes of “Ode to Joy” through thermoelectric signals (Figure S13, Supporting Information). The obtained temperature sensor is a possibility for the future development of a self‐powering electronic organ.

### 2.7 Self‐Powering Wearable Sensing

**Figure 5:**
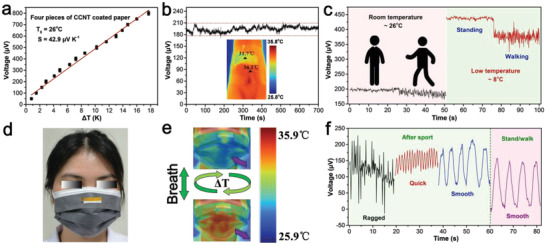
a) Open circuit voltage of thermoelectric generator (TEG) under different temperature difference (*n* = 3). b) The relation between voltage and time when the TEG was connected to a volunteer's wrist (Inset is an infrared image of a TEG immobilized on the wrist). c) Continuous monitoring of the open circuit voltage of the test subject wearing the TEG while standing or walking at room or low temperature. d) Photo of masks used for respiratory rate detection. e) Infrared image of exhalation and inhalation while wearing a mask. f) At room temperature, wearing a mask was used to detect the voltage of breathing afterdoing sports or standing/walking.

As CCNT coated paper based TEG is bio‐safe, we directly installed it on human skin for detailed testing to study its practicability as a wearable electronic product. A flexible TEG was formed by connecting four pieces of 5 × 15 mm CCNT coated papers in series with copper wire. Firstly, the thermoelectric properties were characterized at room temperature of 26 °C and relative humidity of 85%. The infrared imaging picture of TEG was shown in Figure S14 (Supporting Information). By adjusting the temperature of the platform, the temperature difference between the two ends of TEG was changed, and the corresponding thermal voltage was recorded (**Figure** **5**a). According to the voltage–temperature difference curve, the slope of the curve was 42.9 µV K^−1^, corresponding to the Seebeck coefficient of the TEG. To evaluate the self‐powering performance of TEG, it was stuck directly on the wrist of the volunteer. One end of TEG was in direct contact with the skin, and the other end was separated from the skin by an insulating layer. The illustration of Figure 5b was the infrared image of TEG attached to the volunteer wrist, which recorded the actual construction of TEG. The room temperature was 25.3 °C, the temperature difference generated by the wrist was 2.5 K, and the corresponding thermal voltage was about 0.2 mV (Figure S15, Supporting Information). The volunteer was in a static state during the test for 700 s, and the thermal voltage generated by TEG was stable at 0.2 mV and slightly fluctuated (Figure 5b). These results showed that the TEG could stably collect human energy at room temperature and realize self‐powering. Besides, we stuck the coated paper on the outside of the beaker and changed the temperature in the beaker to test the adaptability of thermoelectric paper in hot and cold environments. The internal resistance of coated paper fluctuates in 610–630 Ω with the change of temperature at one end of coated paper, which can be ignored (Figure S16, Supporting Information). It means that TEG can continuously monitor the movement of the human body in indoor activities. As shown in Figure 5c, the output voltage changed with the behavior of the human body at room temperature or low temperature. The maximum output voltage was the same when standing or walking at room temperature, indicating that the indoor airflow was low. The thermal voltage was mainly determined by the temperature difference between the body and ambient temperature. Therefore, when the indoor temperature changed from the room temperature (26 °C) to the low temperature (8 °C), the maximum output voltage would increase as the temperature difference increased. Additionally, when walking, due to the change in airflow, the temperature difference changed regularly, and the output voltage fluctuated within a specific range.

Furthermore, we installed TEG in the mask to realize health monitoring by detecting the respiratory rate of the human body. Embedded one end of TEG into the mask so that it was close to the nostril, and the other end was exposed to the air, as shown in Figure 5d. Figure 5e shows the infrared image of the mask breathing with the human body. When the volunteers inhaled, the temperature at both ends of TEG was close, and the output voltage was small (close to 0 mV). When the volunteer exhaled, the TEG temperature at one end near the nostril rose rapidly, while the temperature at the end exposed to the air remained the same, thus increasing the output voltage. So, when volunteers stood or walked, the respiratory rate was stable, and the generated thermal voltage changed regularly (Figure 5f). After strenuous exercise the volunteers' breathing went through three stages of ragged, quick, and smooth breathing frequency, which led to different thermal voltage signals. It is worth noting that ragged breathing showed irregular thermal voltage signals, which may be due to unsmooth breathing, but one should try our best to avoid it. This product can be applied in many healthcare areas, including hospitals and daily life. For example, due to Covid‐19, we have to wear masks outdoors to prevent infection. Simply embedding the obtained TEG into a mask can monitor whether breathing is normal or not in a self‐powering environment in real‐time through the output voltage.

### 2.8 Self‐Powering Strain Sensing

**Figure 6:**
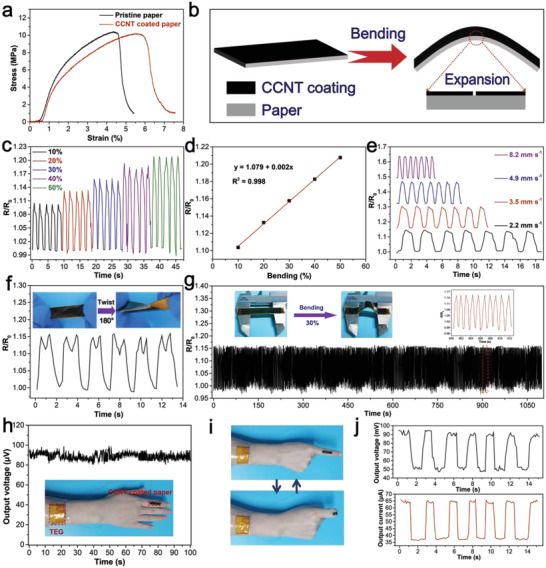
a) Stress–strain curves of the pristine paper and CCNT coated paper. b) Schematic diagram of the bending strain sensing mechanism of the strain sensor. c) Strain sensing behavior at different bending strains. d) The relative resistance changed of the sensor as a function of bending strain. e) Strain sensing behavior at 30% strain at different bending rates. f) Cyclic torsional sensing behavior of the strain sensor at a twist angle of 180°. g) Long‐term durability of the strain sensor for 1000 cycles at 30% strain. h) Schematic illustration of a self‐powering strain sensor attached to the skin and its corresponding output voltage. i) Schematic of a folded finger with self‐powering strain sensors. j) Responses to voltage and current changes when fingers are folded.

CCNT coated paper based TEG not only has a good temperature response but also has the potential as a strain sensor because of its good flexibility. The stress–strain curves of pristine paper and CCNT coated paper were shown in **Figure** **6**a. The results showed that the stress of the coated paper was almost unchanged, but the strain increased from 5.4% to 7.5% after coating with CCNT. This coated paper was then used as a strain sensor, and the sensing mechanism was shown in Figure 6b. When bending to the uncoated end, the coating will be subjected to tensile stress, and the microcracks will spread, resulting in the destruction of the conductive network and the increase of resistance.

Subsequently, the strain and cyclic properties of coated paper under different bending degrees and curvatures were studied to evaluate its applicability in various environments. As the bending degree increased, the relative resistance increased because the tensile stress of the coating increased, which made the network change more serious (Figure 6c). As shown in Figure 6d, there was a high linear relationship between bending strain and relative resistance (*R* ^2^ = 0.998). The coated paper showed good stability and recoverability under different bending curvatures and had typical sensing behavior dependenting on of curvature (Figure 6e). Besides, the strain sensing performance of coated paper at a 180° twist angle was studied (Figure 6f). The repeatable and stable response signal could also be obtained during cyclic torsion. Furthermore, its long‐term sensing performance was examined through 1000 bending cycle tests (Figure 6g). The coated paper kept good relative resistance stability in the bending test. The illustration in Figure 6g was a photo of the cyclic bending process and the sensing behaviors of 10 cyclic bending randomly selected. It can be seen that almost the same bending sensing signals and excellent reproducibility were displayed in each cycle. The results showed that coated paper has good mechanical flexibility and durability, which is very important for wearable sensors in practical applications.

We attached four 5 × 15 mm coated papers in series to volunteers' wrists as power supply and coated papers attached to thumb joints as strain sensors to test the strain sensing performance of the coated paper in a self‐powering environment. A self‐powering strain sensing platform built by us was shown in Figure 6h. Under the steady state, TEG produced a stable output voltage due to the temperature difference between the environment and the skin. When the volunteers' joints repeatedly bend (Figure 6i), the output voltage and current that change regularly were collected through the electrometer. In this case, the output voltage or current signal was shown in a rectangular shape because of the fast response of the voltage or current in the circuit. In addition, the response time of the output voltage was tested to be 0.51 s (Figure S17, Supporting Information), which is faster than some previously reported strain sensors (0.6–0.96 s),^[^ ^42^ ^]^ and has great potential for real‐time monitoring of human motion.

## 3 Conclusions

Noncovalent interactions including *π*–*π* interactions, hydrophobic interactions, and electrostatic attractions occurred between natural derived ChNCs and MWCNT. The high colloidal stability of ChNCs endowed CCNT dispersion with excellent stability and dispersibility, and the dispersion efficiency of ChNCs was as high as 91.1%. ChNCs also were used as adhesive, which made CCNT have good printability and adaptability to various substrates (paper, glass, and PET), thus proving that CCNT dispersion had great application potential as a conductive ink. Compared with other MWCNT‐based conductive inks, CCNT ink promoted the utilization of marine resources, and provided an idea for natural surfactants to disperse MWCNT. Self‐supporting films could be formed from CCNT dispersion by solvent evaporation, and the conductivity was as high as about 1150 S m^−1^. A TEG based on CCNT was prepared by a simple screen‐printing method on paper substrate. The TEG had good biosafety and flexibility, and CCNT ink adhered evenly to the surface and inner upper layers of cellulose paper. CCNT‐based TEG could effectively convert thermal energy into electrical energy, which could produce a maximum output voltage of 0.375 mV at a temperature difference of 12 K. CCNT‐based TEG had the ability of accurate temperature detection and identification and could respond at the minimum temperature difference of 0.7 K. A self‐powering sensor was designed to convert thermal voltage signals into musical notes. Besides, this device exhibited a good Seebeck effect with Seebeck coefficient of 42.9 µV K^−1^. It could be directly installed on the volunteer's arm or in a mask to detect the human body's movement and respiratory rate. Since paper as the substrate provided good flexibility, the relative resistance of TEG remained stable after 1000 bending cycles. The self‐powering strain sensor could sensitively monitor the movement of human joints. In a word, MWCNT‐based conductive ink dispersed by ChNCs has a broad application prospect of self‐powering multifunctional wearable electronics. Improving the thermoelectric performance of CCNT coated paper and assembling into a portable microgenerator should be done in future research.

## 4 Experimental Section

### Materials

Shrimp shells were purchased from Wuhan Hezhong Biochemical Manufacturing Co., Ltd., China. MWCNT was purchased from Nanjing Xianfeng Nano Material Technology Co., Ltd., China. The diameters of MWCNT were 10–20 nm, the lengths were 10–30 µm, and the purity was 95%, according to the supplier. Cellulose paper was provided by Hangzhou General Electric Biotechnology Co. Ltd., China. Acridine orange/ethidium bromide (AO/EB) staining reagents were obtained from Beijing Solarbio Science and Technology Co., Ltd., China. Other chemical reagents were purchased from Aladdin Industrial Corp., China. Milli‐Q Integral Water Purification System produced deionized water.

### Preparation of ChNCs

ChNCs were extracted from shrimp shell by hydrochloric acid hydrolysis. An amount of 20 g of shrimp shell was mixed with 600 mL, 3 mol L^−1^ HCl solutions and reacted at 104 °C for 4 h under strong mechanical stirring. Afterward, deionized water was added to stop the reaction. The suspension was centrifuged at 8000 rpm min^−1^ three times to remove excess HCl. Then, the suspension was dialyzed in deionized water until the pH value of the suspension remained unchanged. Finally, the suspension was freeze‐dried in a vacuum freeze‐dryer (Ningbo Scientz Biotechnology Co., Ltd., China) for 24 h to obtain ChNCs powder for subsequent experiments.

### Preparation of CCNT Dispersions

MWCNT with different amounts were added to 10 mL of 4 wt% ChNCs dispersions, and the mixture was ultrasonicated for 60 min under 70 W power (Xiaomei Ultrasonic Instrument (Kunshan) Co., Ltd., China) to obtain uniform ChNCs/MWCNT (CCNT) dispersion. The sample code was abbreviated as CCNT‐22, CCNT‐27, and CCNT‐33, corresponding to the concentration of MWCNT in CCNT dispersion of 22, 27, and 33 mg mL^−1^, respectively. The CCNT‐33 dispersion was cast in a polystyrene Petri dish and dried in an oven to obtain the CCNT film. CCNT was coated on cellulose paper, glass, or PET by the screen printing.

### Preparation of CCNT Coated Paper Based TEG

Firstly, the concentration of CCNT‐33 ink was adjusted to 0.9, 4.5, 6.3, and 7.2 wt%, respectively, by addition of deionized water. Then, the ink was printed on the surface of cellulose paper by a screen‐printing plate engraved with a 5 × 15 mm rectangular pattern using a brush plate. The flexible TEG was prepared by drying in an oven at 50 °C, connecting several coated papers in series with copper wire, and packaging them with polyimide.

### Characterization of the CCNT Dispersions

The morphology of MWCNT and CCNT was observed using TEM (JEM‐1400 Flash, JEOL Ltd., Japan). A Nano‐ZS instrument (Malvern Instruments Ltd., U.K.) was used to test the zeta potentials and size of the ChNCs and CCNT dispersions. Raman spectroscopy of MWCNT and CCNT was performed using a high‐resolution Raman spectrometer (LabRAM HR Evolution, HORIBA, Japan). The spectrum was recorded from 100 to 3000 cm^−1^. FTIR spectra of MWCNT, ChNCs, and CCNT were measured by using a FTIR spectrometer (UATR Two, PerkinElmer, USA) over the scanning range of 4000–400 cm^−1^. The UV–vis absorption spectra of 0.5 g L^−1^ ChNCs or CCNT dispersions was measured by a UV–vis spectrophotometer (UV‐2550, Shimadzu Instrument Ltd., Suzhou, China) ranging from 200 to 800 nm. Rheological properties of ChNCs and CCNT ink were measured by rotary rheometer (Discovery HR‐20, TA Instruments, USA) at room temperature. The parallel plate model was used for testing, and the plate diameter and gap was 20 and 0.6 mm, respectively. The apparent viscosity was measured at a shear rate of 0.01 to 100 1 s^−1^. An oscillating time scanning experiment evaluated the viscoelasticity of ChNCs and CCNT ink. Measurements were made at a constant scanning frequency of 1 Hz and an oscillating strain of 0.1% to 1000%. TG analysis (Mettler Toledo, Switzerland) was used to determine the thermal stability of MWCNT dispersed by ChNCs. The sample was heated from 30 °C to 800 °C in a nitrogen atmosphere at a heating rate of 10 °C min^−1^. The contact angle of CCNT ink (droplet volume was 5 µL) on different substrates was measured by a contact angle instrument (DSA100, Kruss Ltd., Germany) at room temperature.

The electrical conductivity (*σ*) of CCNT film or CCNT coated paper was calculated using the following equation:

(1) $$\sigma \left(Sm^{-1}\right)=\frac{l}{\mathrm{RS}}$$

where *l* (m) is the length, *R* (Ω) is the resistance, and *S* (m^2^) is the cross‐section area of the film or coating.

MWCNT and dispersants, i.e., PVP, CNC, and ChNCs, were mixed with deionized water to a certain volume. The weight ratio of dispersant and MWCNT was kept at 11:9. The MWCNT dispersions were prepared by an ultrasonic cell grinder at 70 W power ultrasonic for 1 h. Then, the dispersion was centrifuged at 4000 rpm for 10 min to remove the MWCNT aggregates. The ability of dispersant to stably disperse MWCNT was measured by evaluating the dispersion efficiency. The equation of dispersion efficiency was as follows:

(2) $$\mathrm{Dispersion}\;\mathrm{efficiency}\left(\%\right)=\frac{C}{C_{0}}\times 100\%$$

where *C* (mg mL^−1^) is the concentration of MWCNT in the dispersion after centrifugation (measured by UV–vis absorption spectra at 260 nm); *C*_0_ (mg mL^−1^) is the concentration of MWCNT in the initial dispersion before centrifugation (the initial concentration was 33 mg mL^−1^).

### Biocompatibility Evaluation of CCNT Ink Coated Paper

The biological toxicity of CCNT ink coated paper was evaluated with wheat as a plant model. Wheat seeds were firstly soaked in deionized water for 24 h. Then transfer the seeds to a Petri dish containing CCNT ink coated paper for germination and growth, and pristine paper was used as the control group. The survival rate, height, and average root length of wheat seedlings were counted after 10 days of cultivation.

The toxicity of CCNT coated paper extracts with different concentrations (20%, 40%, 60%, 80%, and 100%) to L929 cells was determined by the CCK‐8 method. Firstly, L929 cells were incubated in the culture medium in an incubator with 37 °C and 5% CO_2_. Then, these cells were inoculated on a 96‐well plate at a density of 4 × 10^4^ cells mL^−1^ and incubated in an incubator (37 °C, 5% CO_2_). After 24 h, the cells adhered to the wall, and samples with different concentrations were added to the cells, respectively, taking the culture medium as the control. The cells were continuously incubated for 24 or 48 h. Subsequently, 10 µL CCK‐8 solution was added to each well and incubated at 37 °C for 4 h. The absorbance was measured at 450 nm using a microplate reader (Bio‐Tek, Hercules, USA). The equation of cell viability was as follows:

(3) $$\mathrm{Cell}\;\mathrm{viability}\left(\%\right)=\frac{A_{\mathrm{sample}}}{A_{\mathrm{control}}}\times 100\%$$

where *A*_sample_ is the absorbance of the sample groups; *A*_control_ is the absorbance of the control group.

For the live/dead assay, L929 cells were incubated in different concentrations (20%, 40%, 60%, 80%, and 100%) of CCNT coated paper for 24 or 48 h, and then the culture medium was carefully removed. Then cells were stained with AO/EB for 10 min. After washing with PBS to remove excess stain, the cells were covered with PBS. The cells were observed by fluorescence microscope (XDY‐2, Guangzhou Liss Optical Instrument Ltd., China), and images were taken in a random field of vision.

### Characterization of CCNT Coated Paper Based TEG

The surface and cross‐sectional morphologies of raw paper and CCNT coated paper were observed by a field emission SEM (Zeiss Sigma 300). The homemade platform was combined with the electrometer (Keithley 6514) to collect the output voltage data. The homemade cold and hot platform was used to control the temperature of the cold and hot ends of coated paper. Briefly, the coated paper with a size of 5 × 15 mm was placed on a cold and hot platform, with one end for heating and the other end for cooling to generate a temperature difference. The Seebeck coefficient was obtained from the slope of the voltage and temperature difference curve. The infrared imager (TIS55, Fluke Electronic Instrument Ltd., USA) was used to record the temperature and image of TEG in real time. Laser thermal diffusion/thermal conductivity tester (LFA 1000, Linseis, Germany) was used to test the thermal conductivity of CCNT coated paper. The strain property of precision paper and coated paper was measured by a universal testing machine (AGS‐X, Shimadzu, Japan). The resistance, voltage, and other signals of the sensor were recorded with Keithley 6514 electrometer. All volunteers gave written informed consent before participating in the experiment.

### Statistical Analysis

Experimental data were processed and expressed using mean values ± standard deviation (SD). Statistical comparisons of the data were conducted using GraphPad Prism 7 (GraphPad Software, USA). The between‐group differences were analyzed using a one‐way analysis of variance (ANOVA). Differences were considered statistically significant at *p* < 0.05.

## Conflict of Interest

The authors declare no conflict of interest.

## Supporting information

Supporting Information

## Data Availability

The data that support the findings of this study are available from the corresponding author upon reasonable request.
