# Supplementary material for: Carbon Nanotube Ink Dispersed by Chitin Nanocrystals for Thermoelectric Converter for Self‐Powering Multifunctional Wearable Electronics
Source: Adv Sci (Weinh). 2022 Oct 6;9(33):2204675. doi: 10.1002/advs.202204675 (PMC9685456; doi:10.1002/advs.202204675)
Supplement: Supplementary file 1 — Supporting Information [file ADVS-9-2204675-s001.pdf]

ADVANCED SCIENCE
Open Access

Supporting Information

for *Adv. Sci.*, DOI 10.1002/advs.202204675

Carbon Nanotube Ink Dispersed by Chitin Nanocrystals for Thermoelectric Converter for Self-Powering Multifunctional Wearable Electronics

*Yunqing He, Xiaoying Lin, Yue Feng, Binghong Luo and Mingxian Liu**

Carbon Nanotube Ink Dispersed by Chitin Nanocrystals for Thermoelectric Converter for Self-Powering Multifunctional Wearable Electronics

*Yunqing He, Xiaoying Lin, Yue Feng, Binghong Luo, Mingxian Liu**

Department of Materials Science and Engineering, College of Chemistry and Materials Science, Jinan University, Guangzhou 511443, China.

Email: liumx@jnu.edu.cn

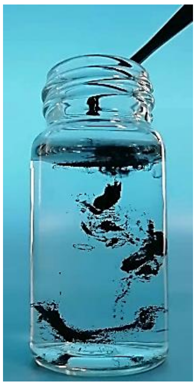

Figure S1. Drops of sonicated bare MWCNT in water.

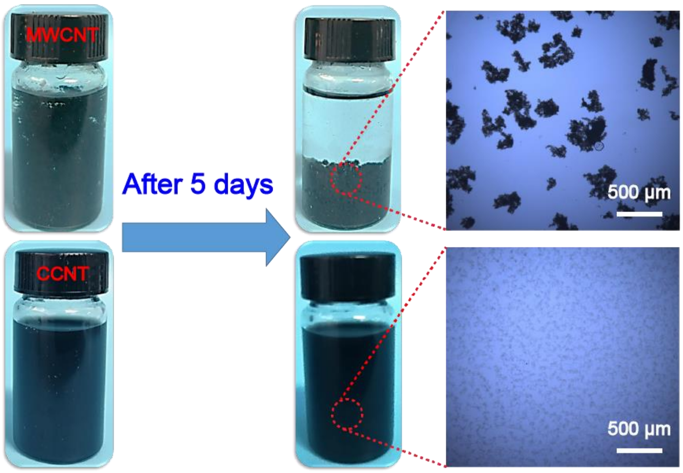

Figure S2. The digital picture and the optical microscope images of MWCNT aqueous solution (0.5 wt%) and CCNT dispersions (0.5 wt%).

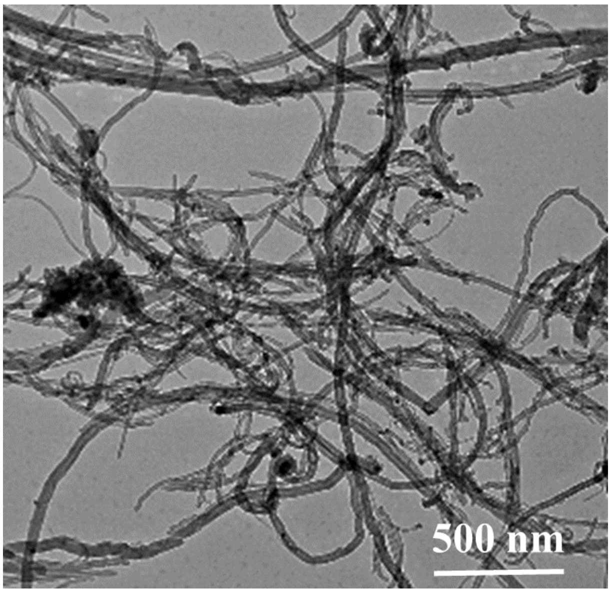

Figure S3. The TEM image of MWCNT aqueous solution.

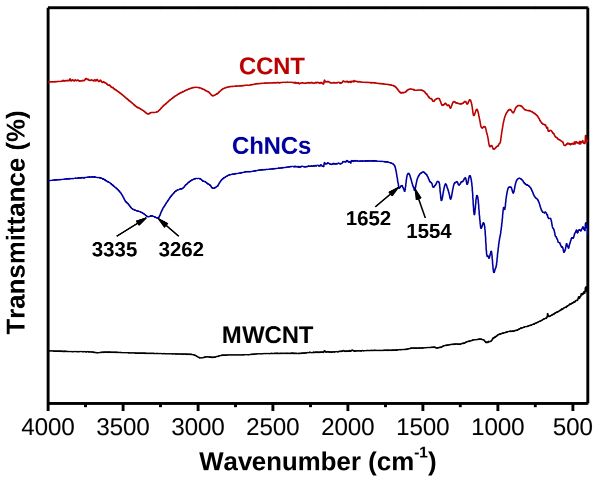

Figure S4. FTIR spectra of MWCNT, ChNCs and CCNT.

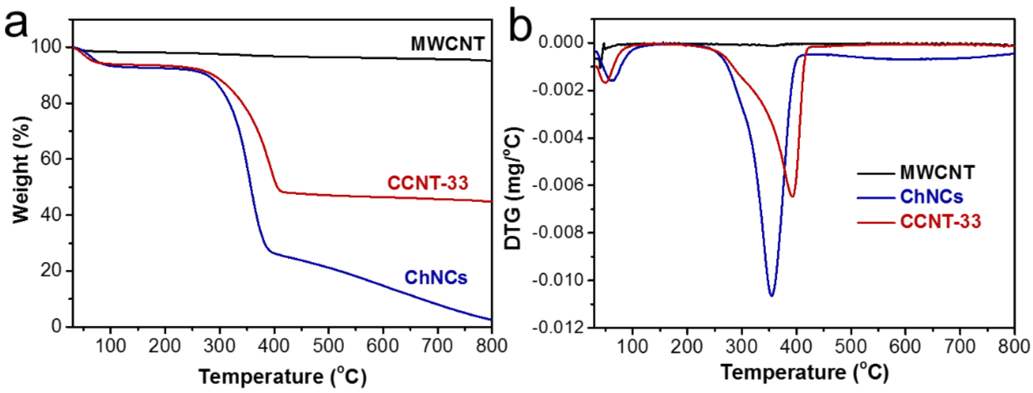

Figure S5. (a) TG and (b) DTG curves of MWCNT, ChNCs and CCNT-33.

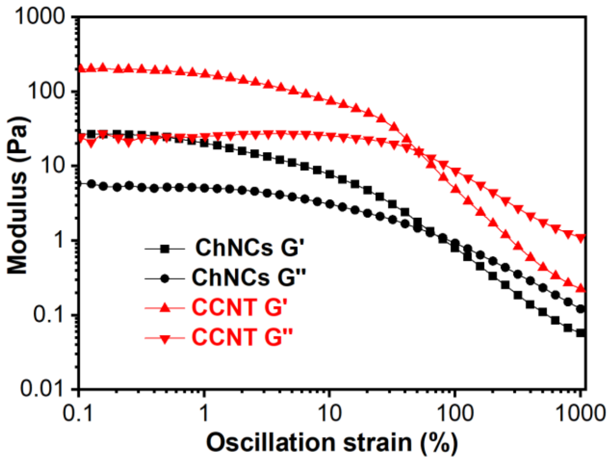

Figure S6. Storage and loss modulus of strain for ChNCs and CCNT.

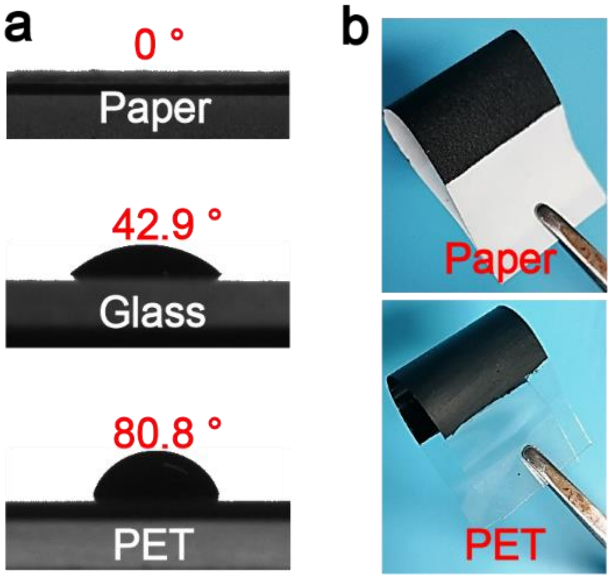

Figure S7. (a) Images of contact angles for CCNT ink on different substrates; (b) The printed coating can be bent following the flexible substrates.

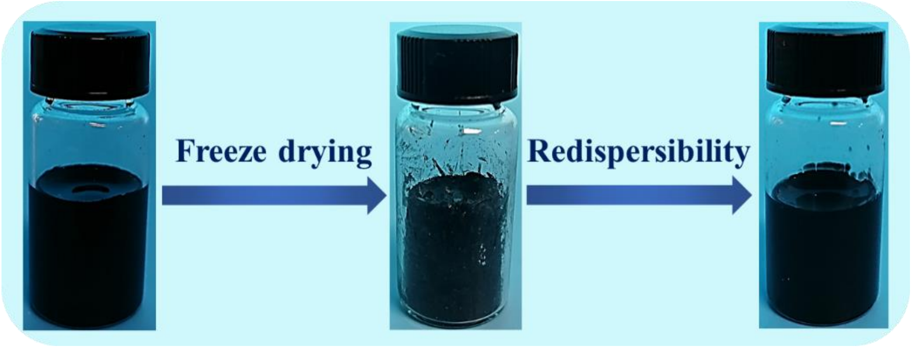

Figure S8. Flow chart of the dispersibility of CCNT ink.

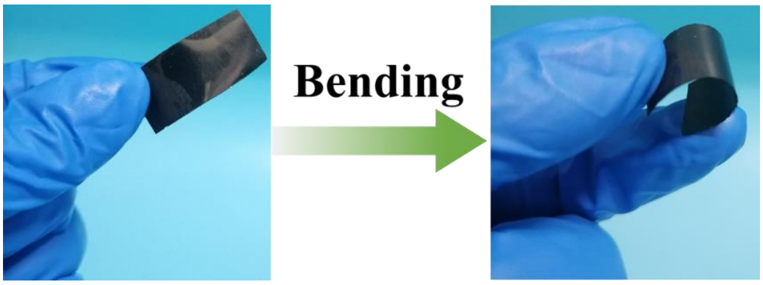

Figure S9. Digital picture of film-forming properties CCNT ink.

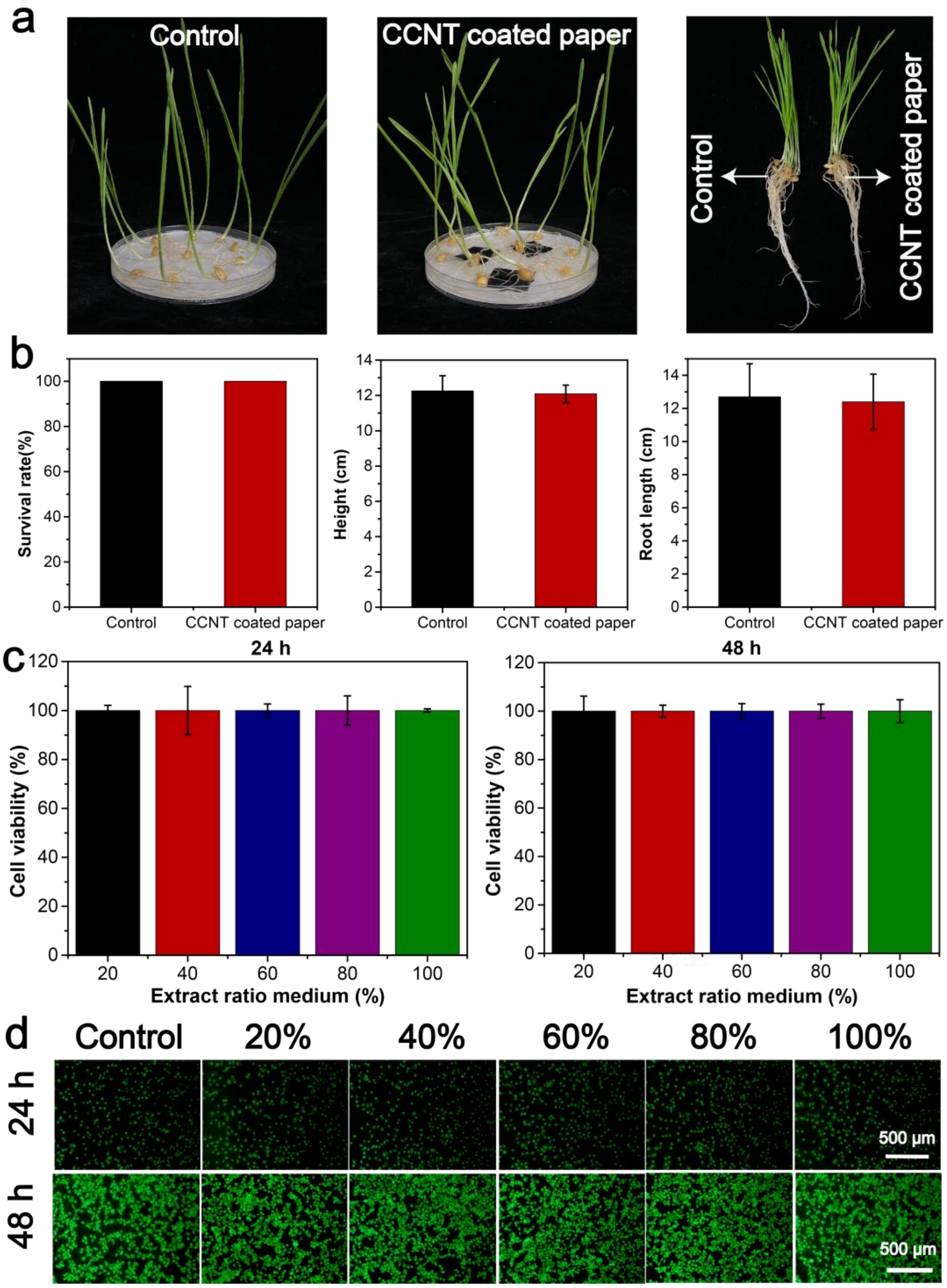

Figure S10. a) Photos, b) survival rate, height, and average root length of wheat seedlings after culturing for 10 days (n = 10). c) The viability of L929 cells in the culture medium containing coated paper extract with different concentrations by CCK-8. d) AO/EB (live/dead) staining images of L929 cells cultured for 24 and 48 h (n = 3).

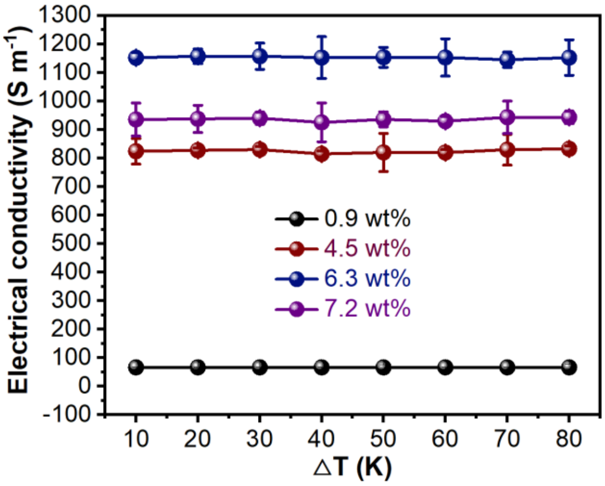

Figure S11. Electrical conductivity of TEG coating prepared by different amounts of CCNT.

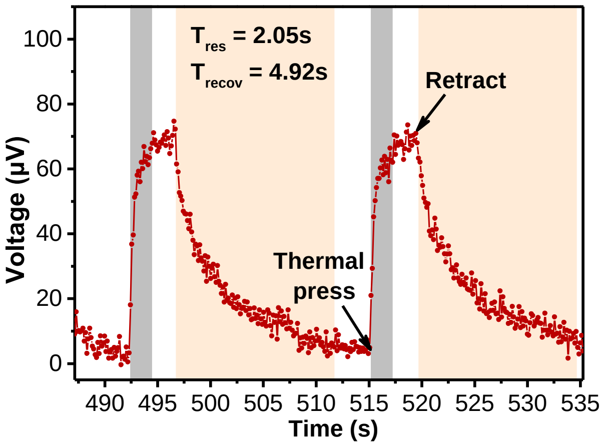

Figure S12. The response and recovery response time of temperature sensing.

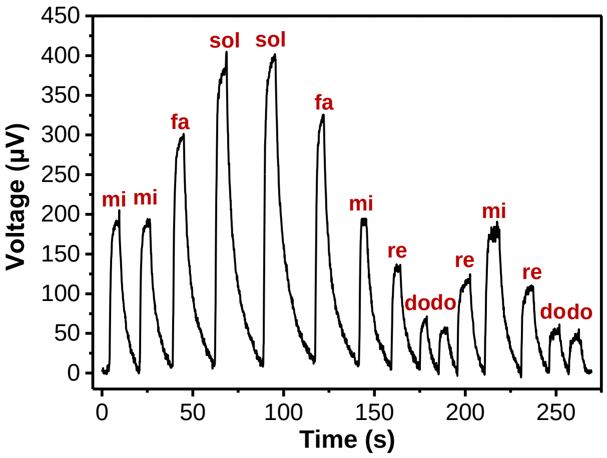

Figure S13. The self-powered temperature sensor converts the voltage generated by the ΔT into musical notes, taking "Ode to Joy" as an example.

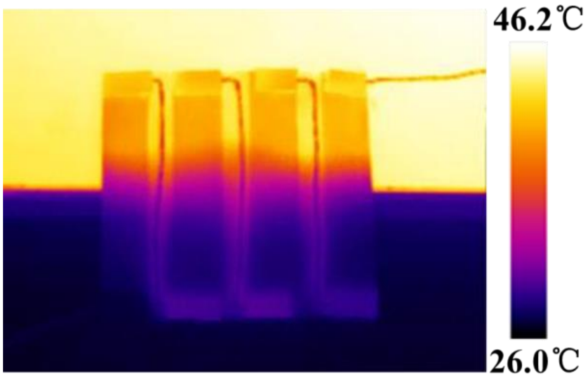

Figure S14. Infrared thermal imaging image of paper-based TEG under ΔT system**.**

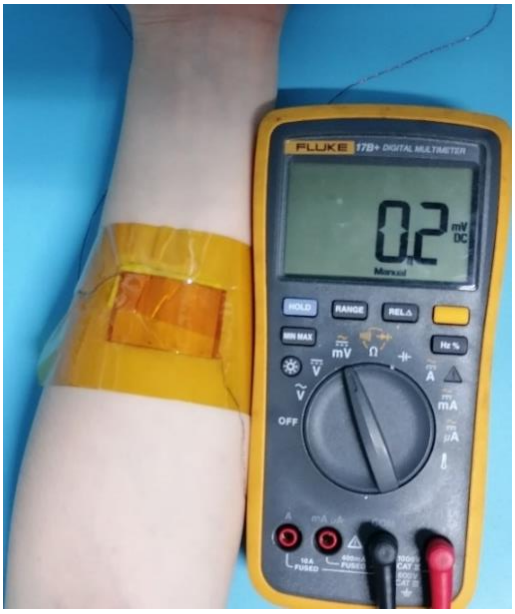

Figure S15. The photo of output voltage of the TEG fixed on the wrist at room temperature.

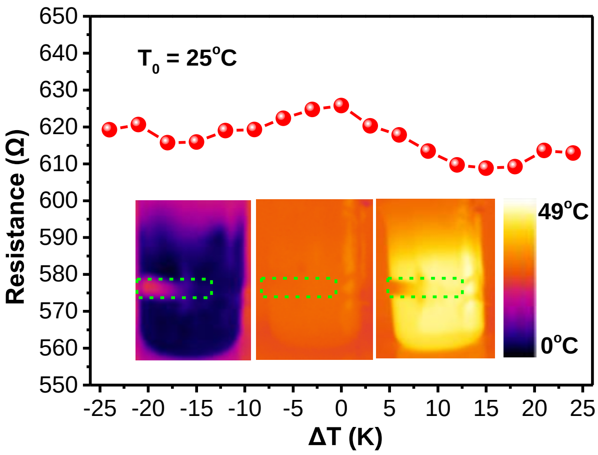

Figure S16. Resistance change of paper based TEG under different temperature differences.

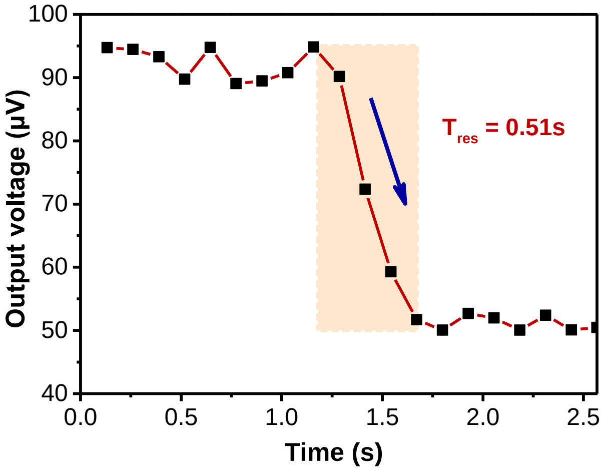

Figure S17. A magnified image of the voltage response time when the sensor is stretched.

Table S1. Comparison of the ZT of reported CNT based thermoelectric materials and CCNT coated paper at room temperature (299 K).

Materials	Method	ZT	Reference
CCNT coated paper	Screen-printing	0.0041	This work
SWCNT/cellulose acetate composite films	Bar-coating process	0.0011	[40] a)
CNT/polyaniline composites	In situ polymerization	0.0008	[40] b)
CNT/PANI network composites	Immersing	0.0022	[40] c)
MWCNTs paper	A vacuum filtration	0.0012	[41]
